# Retraction: Endothelin-1 Inhibits Prolyl Hydroxylase Domain 2 to Activate Hypoxia-Inducible Factor-1α in Melanoma Cells

**DOI:** 10.1371/journal.pone.0315282

**Published:** 2024-12-05

**Authors:** The PLOS ONE Editors

After this article [1] was published, concerns were raised about Figs 1–5. Specifically,

- There appear to be similarities between bands within numerous panels presented in:
  - Figs 1B and 1D
  - Fig 2A
  - Fig 3A
  - Fig 4B
  - Fig 5A

- There appear to be similarities between bands between numerous panels presented in:
  - Fig 1A and Fig 1B
  - Fig 1A and Fig 3C
  - Fig 3A and Fig 3C
  - Fig 3A and Fig 4A

- There appear to be irregularities in the background directly surrounding the band in lane 2 of the Fig 4B HIF-1α panel.

The corresponding author disagreed with the journal’s observations but did not provide the underlying raw image data. PLOS remains concerned that the areas remain more similar than would be expected from independent results which, in the absence of the underlying data, cannot be resolved.

In light of the above concerns, which question the reliability and integrity of the reported results, the *PLOS ONE* Editors retract this article.

AB did not agree with the retraction and stands by the article’s findings. FS, LR, MDD, VDC, MRN, and PGN either did not respond directly or could not be reached.
